# Interobserver and sequence variability in the delineation of pelvic organs at risk on magnetic resonance images

**DOI:** 10.2478/raon-2025-0006

**Published:** 2025-01-22

**Authors:** Wanjia Zheng, Xin Yang, Zesen Cheng, Jinxing Lian, Enting Li, Shaoling Mo, Yimei Liu, Sijuan Huang

**Affiliations:** 1 State Key Laboratory of Oncology in South China, Guangdong Provincial Clinical Research Center for Cancer, Sun Yat-Sen University Cancer Center, Guangzhou, Guangdong Province, China; 2 Department of Radiation Oncology, Southern Theater Air Force Hospital of the People’s Liberation Army, Guangzhou, Guangdong Province, China; 3 Guangdong Esophageal Cancer Institute, Guangzhou, Guangdong Province, China; 4 United Laboratory of Frontier Radiotherapy Technology of Sun Yat-sen University & Chinese Academy of Sciences Ion Medical Technology Co. Ltd, Guangzhou, Guangdong Province, China; 5 School of Electronic and Computer Engineering, Peking University, Shenzhen, Guangdong province, China; 6 Department of Radiation Oncology, The First Affiliated Hospital of Guangzhou University of Chinese Medicine, Guangzhou, Guangdong province, China; 7 Department of Radiology, The Eighth Affiliated Hospital of Sun Yat-sen University, Shenzhen, Guangdong province, China; 8 Department of Radiation Oncology, The First People’s Hospital of Foshan, Foshan, Guangdong province, China

**Keywords:** MRI, multiple sequences, variability, automatic segmentation

## Abstract

**Background:**

This study evaluates the contouring variability among observers using MR images reconstructed by different sequences and quantifies the differences of automatic segmentation models for different sequences.

**Patients and methods:**

Eighty-three patients with pelvic tumors underwent T1-weighted image (T1WI), contrast enhanced Dixon T1-weighted (T1dixonc), and T2-weighted image (T2WI) MR imaging on a simulator. Two observers performed manual delineation of the bladder, anal canal, rectum, and femoral heads on all images. Contour differences were used to analyze the interobserver and intersequence variability. A single-sequence automatic segmentation network was established using the U-Net network, and the segmentation results were analyzed.

**Results:**

Variability analysis among observers showed that the bladder, rectum, and left femoral head on T1WI yielded the highest dice similarity coefficient (DSC) and the lowest 95% Hausdorff distance (HD) (all three sequences). Regarding sequence variability analysis for the same observer, the difference between T1WI and T2WI was the smallest. The DSC of the bladder, rectum, and femoral heads exceeded 0.88 for T1WI–T2WI. The differences between automatic segmentations and manual delineations were minimal on T2WI. The averaged DSC of automatic and manual segmentation of all organs on T2WI exceeded 0.81, and the averaged 95% HD value was lower than 7 mm. Similarly, the sequence variability analysis of automatic segmentation indicates that the automatic segmentation differences between T2WI and T1WI are minimal.

**Conclusions:**

T1WI and T2WI yielded better results in manual delineation and automatic segmentation, respectively. The analysis of variability among three sequences indicates that the yielded good similarity outcomes between the T1WI and T2WI cases in manual and automatic segmentation. We infer that the T1WI and T2WI (or their combination) can be used for MR-only radiation therapy.

## Introduction

Adaptive radiotherapy (ART) is useful for detecting changes in the position, shape, size, and other characteristics of the target and organs at risk (OARs) during radiotherapy. Appropriate adjustments to the treatment plan can improve the dose consistency and protect normal tissues.^1,2^

Accurate delineation of targets and OARs is a key aspect of the ART process. Compared with computed tomography (CT), magnetic resonance imaging (MRI) has the advantage of accurate softtissue contrast and does not produce additional ionizing radiation doses.^3,4^ Some studies^5,6^ have pointed out that compared with CT, the volumes of tumor targets and OARs delineated on magnetic resonance imaging is significantly reduced such that the tumor can receive a higher dose. Simultaneously, the protection of normal tissues can be equivalent to or even better than CT.^7,8^ MRenhanced soft tissue not only improves the positioning accuracy of patients before radiotherapy but also improves the positioning of tumors and normal tissue during real-time imaging during treatment, thus making dose delivery more accurate.^9,10^ Vestergaard *et al*. found that re-optimized ART for MRI-guided bladder cancer treatment has considerable sparing potential for normal tissues.^11^

For lengthy MR scans, only one sequence is used for radiotherapy. Most studies have used T2-weighted and related sequences to delineate the tumor volume, but there is no consensus on which sequence should be used to delineate OARs.^12^ However, in some studies, experts recommended the use of the extended T2-weighted sequence to delineate the target and OARs.^13^

Therefore, in this study, we performed manual delineation of OARs on images reconstructed using three sequences (T1-weighted image [T1WI], contrast enhanced Dixon T1-weighted [T1dixonc], T2-weighted image [T2WI]), which are commonly used in MRI simulators, to analyze interobserver and intersequence variability. Simultaneously, we automatically segmented the images obtained using these three sequences to observe the stability of OARs in automatic segmentation.

## Patients and methods

### MR image acquisition

This study enrolled 83 patients diagnosed with cervical cancer and treated at the SUSYCC Cancer Center between March 2017 and December 2018. The median age of patients at the time of scanning was 54 years (22–82 years). MR images collected from 54 patients were used as the training cohort to build a single-sequence, automatic segmentation model, and images from the remaining 29 patients were used to analyze the variability of the manual segmentation outcomes of the OARs.

Patients were scanned in supine positions in a vacuum bag with their hands raised. MRI scans were conducted using a 70-cm bore Ingenia 3.0 T scanner (Philips, Netherlands), with a slice thickness of 3 mm. Three MRI sequences were selected and imported into the Monaco Planning System. The selected sequences and their respective parameters were as follows: T1WI (repetition time [TR]: 710 ms; echo time [TE]: 15 ms), T1dixonc (TR: 5.5 ms; TE: 3.7 ms), and T2WI (TR: 6088 ms; TE: 105 ms).

The basic data had been submitted to a public Research Data Deposit (RDD) platform (www.researchdata.org.cn), with an approval RDD number as RDDA2021001910.

### OAR contouring

Due to the limited scanning range, this comparative study is limited to organs with complete contours within the image. According to the Radiation Therapy Oncology Group^14^ and based on the delineated guidelines and clinical requirements for the female’s normal pelvic tissue, bladder, rectum, anal canal, and femoral heads (left and right) were delineated on the three MRI sequences. Delineation of the rectum began at the junction of the third bone plane with the sigmoid colon and ended at the junction with the anal canal above the anorectal line. The delineation of the anal canal started at the anorectal line and ended at the anus. The bladder included all the bladder walls and their contents. Manual delineation of all organs were independently completed by two pelvic oncologists (R1 and R2) with more than five experience of career and were handed over to the same more senior pelvic oncologist for independent validation of all contours.

### Automatic segmentation

There were two cohorts: a training cohort and a testing cohort. The training cohort includes 54 samples, and the testing cohort includes 29 samples. All samples encompass three sequences and are annotated with contours for both R1 and R2.

The training cohort was used for U-Net^15^ networks for automatic segmentation, while the testing cohort was used to evaluate the network’s automatic segmentation performance.

The contour of OARs includes the bladder, rectum, anal canal, and femoral heads (left and right).

### OAR evaluation

The dice similarity coefficient (DSC) and Hausdorff distance (HD) are the commonly used evaluation indicators to quantify contouring differences.^16^ In this study, the DSC and 95% HD were used to assess volume- and distance-related differences, respectively.

1 $$D(A,B)=\frac{2|A\cap B|}{\left|A|+|B\right|}$$

where A and B represent two different contour volumes, and the DSC value ranges from zero to one. DSC values > 0.7 mean that the two contours coincide well^17^, and a DSC value of one indicates that the two contours coincide completely.

The directed HD orientation from X to Y is the maximum distance from all the points on X to the closest point on Y.


2 $${\vec{d}}_{H}(X,Y)=\;\overset{\max}{x\in X}\overset{\min}{y\in Y}d(x,y)$$

The (undirected) HD is the maximum of the two directed Hausdorff measures.


3 $$d_{H}(X,Y)=\max\left\{{\vec{d}}_{H}(X,Y),{\vec{d}}_{H}(Y,X)\right\}$$

The 95% HD value can be used to eliminate the influence associated with the elimination of a small part of an inaccurate segmentation on the overall segmentation quality evaluation.^18^ The undirected 95% HD is defined as,

4 $$d_{H,95\%}(X,Y)=\frac{{\vec{d}}_{H,95\%}(X,Y)+{\vec{d}}_{H,95\%}(X,Y)}{2}$$

A lower 95% HD value indicates a smaller difference between the two contours.

All data were analyzed using SPSS (version 25.0; SPSS Inc., Chicago, Illinois, USA). The Wilcoxon rank-sum test was used to compare the results between the two observers. The intraclass correlation coefficient (ICC; two-way random method and absolute agreement for single measures) was used to measure the volume consistency between the two observers and among different sequences. A p-value of <0.05 was considered statistically significant. An ICC greater than 0.75 indicated a good correlation.^19^

## Results

### Interobserver variability

**FIGURE. 1:**
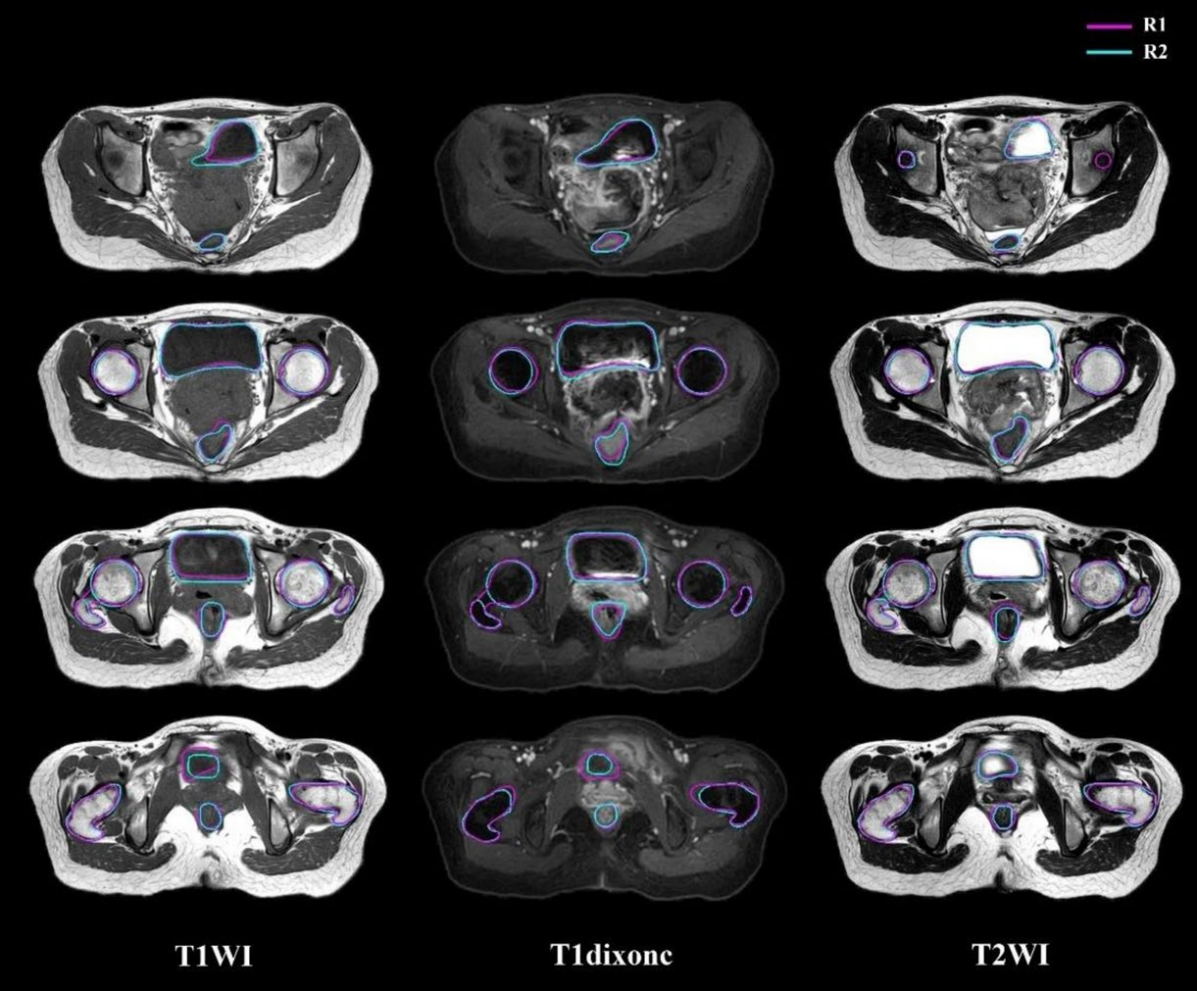
Delineation results on T1WI, T1dixonc, and T2WI performed by the two observers (magenta line: R1; blue line: R2)

**FIGURE. 2:**
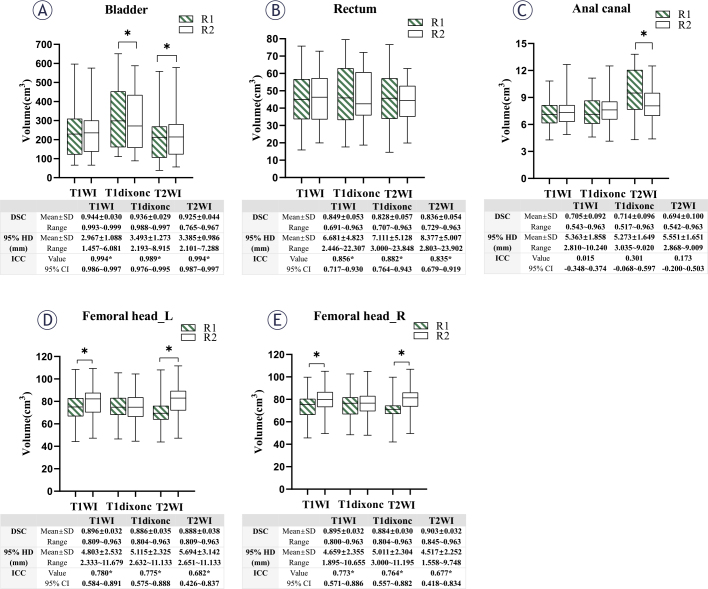
Summary boxplots of the contour volumes and interobserver variability. DSC = dice similarity coefficient; HD = Hausdorff distance; ICC = intraclass correlation coefficient

Figure 1 and Figure 2 shows example and results of the delineation performed by the two observers, respectively. All organs delineated by the two observers yielded the smallest average volume differences on T1WI, except for the left and right femoral heads. The volume correlation outcomes for the bladder and femoral heads showed that T1WI yielded the maximum correlation (all ICC > 0.75). The ICC of the rectum (0.882) obtained by the two observers on T1dixonc was higher than those of the other sequences. The correlation coefficient analysis of the anal canal volume showed that the two observers yielded poor correlation for the anal canal, with ICC < 0.45 on all three sequences. A detailed statistical volume comparison is presented in Supplementary Table 1. Compared with T1dixonc and T2WI, the DSC and 95% HD of the bladder, rectum, and left femoral head were improved on T1WI. The DSC (0.714) and 95% HD (5.273 mm) of the anal canal delineated by the two observers on T1dixonc were better than those on the other sequences. The highest DSC (0.903) and the lowest 95% HD (4.517 mm) of the right femoral head were observed on T2WI.

**TABLE 1.** The dice similarity coefficient (DSC) and 95% Hausdorff distance (HD) (mm) of OARs based on different MR sequences for R2 (mean ± SD)

	OARs	T1WI–T1dixonc	T1WI–T2WI	T2WI–T1dixonc
**DSC**	Bladder	0.877 ± 0.079	0.920 ± 0.038	0.884 ± 0.073
	Rectum	0.842 ± 0.064	0.883 ± 0.049	0.809 ± 0.050
	Anal canal	0.705 ± 0.166	0.760 ± 0.077	0.733 ± 0.125
	Femoral head _L	0.905 ± 0.062	0.952 ± 0.027	0.905 ± 0.055
	Femoral head _R	0.904 ± 0.050	0.959 ± 0.026	0.906 ± 0.049
**95% HD (mm)**	Bladder	6.427 ± 4.360	4.742 ± 1.574	7.092 ± 5.363
	Rectum	5.260 ± 2.934	3.408 ± 1.484	5.953 ± 3.153
	Anal canal	4.732 ± 2.398	4.076 ± 1.375	4.468 ± 2.144
	Femoral head _L	3.811 ± 1.550	2.607 ± 1.405	3.994 ± 1.596
	Femoral head _R	4.027 ± 1.275	1.181 ± 1.171	3.682 ± 1.654

1 L = left; OARs = organs at risk; R = right; T1dixonc = contrast enhanced Dixon T1-weighted; T1WI = T1-weighted; T2WI = T2-weighted

In conclusion, the contours delineated by the two observers on T1WI yielded smaller variations in the most organs.

### Intersequence variability

The volume analysis results of R2 for the contour delineations (images reconstructed Supplementary Table 1. No statistically significant differencs were found in the volume variation of the rectum and right femoral head between T1WI and T2WI. However, there were significant differences between the three sequences in the delineation of the bladder and left femoral head (all p < 0.001).

In the comparison of ICCs between the sequences es Supplementary Table 2, T1WI–T2WI demonstrated an improvement in the correlation of volume compared with the respective correlations of T1WI–T1dixonc and T1dixonc–T2WI (all p 「 0.001). Except for the anal canal (ICC, 0.614; p < 0.001), the ICC between T1WI and T2WI was greater than 0976 (all p < 0.001). The DSC was improved, and 95 HD was reduced in the T1WI–T2WI case compared with the respective values on T1WI–T1dixonc and T2WI–T1dixonc for all OARs. The DSC on T1W T2WI exceeded 0.88 for the bladder, rectum, a femoral heads, and the DSC of the anal canal exceeded 0.75 (Table 1).

**TABLE 2.** The dice similarity coefficient (DSC) and 95% Hausdorff distance (HD) (mm) for different MR sequences of automatic segmentation (mean ± SD)

		Bladder	Rectum	Anal canal	Femoral head _L	Femoral head _R
**DSC**	T1WI- T1dixonc	0.789±0.096	0.686±0.111	0.691±0.121	0.865±0.083	0.876±0.037
	T1WI- T2WI	0.854±0.101	0.784±0.105	0.707±0.087	0.908±0.091	0.924±0.030
	T2WI- T1dixonc	0.756±0.130	0.860±0.912	0.709±0.134	0.891±0.032	0.892±0.037
**95% HD (mm)**	T1WI- T1dixonc	18.079±12.095	9.702±10.940	4.810±2.170	4.300±2.027	4.678±1.793
	T1WI- T2WI	12.459±11.094	7.978±10.469	4.826±2.361	3.362±2.441	3.188±1.413
	T2WI- T1dixonc	17.711±9.049	7.433±3.907	4.478±1.744	3.769±1.204	3.616±1.095

1 L = left; R = right; T1dixonc = contrast enhanced Dixon T1-weighted; T1WI = T1-weighted; T2WI = T2-weighted

The results summarized above show that when the same observer used different sequences for delineation, the similarities between T1WI and T2WI were more than those of other sequence combinations.

### Automatic segmentation

**FIGURE. 3:**
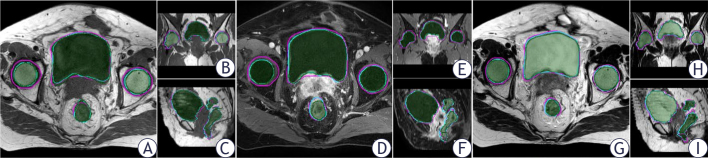
Comparison example between automatic and manual segmentation results in the axial, coronal, and sagittal views on T1WI **(A–C)**, T1dixonc **(D–F)**, and T2WI **(G–I)**. (Magenta line: R1; blue line: R2; and automatic segmentation: shaded green)

Figure 3 shows example of the delineation performed by automatic and manual segmentation. The rectum volumes obtained from automatic segmentation and human observer delineation were significantly different among the three sequences (R1-Auto: all p < 0.04; R2-Auto: all p < 0.03). On T2WI, the volume correlations between automatic and manual segmentations of the bladder and right femoral head were 0.983 (T1WI = 0.933 and T1dixonc = 0.956) and 0.694 (T1WI = 0.673 and T1dixonc = 0.631), respectively Supplementary Table 3. Except for the rectum (T1WI = 0.739 and T2WI = 0.725), the best DSC outcomes of other organs on T2WI were obtained by R1 using automatic segmentation. The lowest 95% HD values of the bladder and right femoral head on T2WI were obtained by R1 using automatic segmentation, and the lowest 95% HD values of the rectum and left femoral head were found on T1dixonc. The best DSC and 95% HD outcomes of all organs were obtained by R2 and automatic segmentation on T2WI Supplementary Table 4.

We analyzed the volume of OARs, volumetric ICC, DSC, and 95% HD to observe the variability in automatic segmentation results among different sequences. There were no significant differences in the volumes of the bladder, rectum, or anal canal among the three sequences. The volumetric ICC between T1WI and T2WI exceeded 0.8 in the bladder, rectum, and right femoral head cases. However, the highest ICC between T2WI and T1dixonc was observed for the anal canal and left femoral head Supplementary Table 5. The greatest DSC and lowest 95% HD values of the bladder and femoral heads were observed in the T1WI–T2WI case compared with those obtained in the T1WI– T1dixonc and T2WI–T1dixonc cases. Compared with T1WI–T2WI and T1WI–T1dixonc, the highest DSC and lowest 95% HD of the rectum and anal canal were found between T2WI and T1dixonc (Table 2).

## Discussion

Although some reports have stated that various MR sequence imaging techniques should be used to assist the positioning or delineation of the target and OARs, no study has identified the optimal sequence for pelvic tumor localization and delineation of the target and OARs. A previous report suggested that better anatomical definition can be achieved with T1-weighted images.^20^ The results of this study indicated that T1WI outperformed the other two sequences in terms of volumetric ICC, DSC, and 95% HD values of the bladder, rectum, and femoral heads, this suggests that the delineated of bladder by the two observers exhibited the least interobserver variability on T1WI. This may be attributed to signal differences in the different sequences of MR images of the bladder. The bladder and urine yielded low signals on T1WI, whereas the surrounding muscles yielded high signals. Compared with T1-weighting, the bladder wall on T2WI without contrast imaging only yielded the muscular layer.^21^ Because of the bright urine observed on T2WI, the filled bladder demonstrated significant contrast with the surrounding muscles, which is beneficial for delineation.^22^ However, the bright urine signal obscured the signal from the urothelium and lamina propria and resulted in an inaccurate measurement of bladder thickness and tumor dimensions on T2-weighted MR images compared with T1-weighted MR images.^21^ The rectal wall had a uniformly low signal on T1WI; this yielded a significant contrast with the surrounding fat layer and is beneficial for delineating the rectum.^23^ Similarly, in this study, the lowest variability between the two observers was observed for the rectum delineated on T1WI (DSC = 0.849). For the delineation of femoral heads, T1WI showed a higher signal intensity than T1dixonc. We found that the interobserver variability in the delineation of the femoral heads on T1WI were less than that on T1dixonc. In terms of variability between sequences, we compared the delineation results of different organs as assessed by a single observer and found that the results on T1WI and T2WI were the closest.

When implementing MRI–ART, the acquisition time of MR images and the optimization of radiotherapy plans are extremely time-consuming. Accurate contour delineation is the most time-consuming and essential step in radiotherapy planning. Several studies have shown that automatic segmentation saves time in real-time planning and reduces inter- and intra–observer variability.^24^ Therefore, we investigated the differences among three sequences in automatic segmentation using MR images obtained from 29 patients as the test set and compared the results obtained using manual and automatic contouring.

R1 and the automatic segmentation model obtained an optimal DSC and 95% HD on T2WI. Except for the anal canal, the DSC of the bladder, rectum, and femoral heads were greater than 0.7. The DSC of the anal canal on all three sequences were greater than 0.6, with the highest DSC obtained on T2WI (0.669). R2 obtained the best DSC and 95% HD values when compared with the automatic segmentation model for all organs on T2WI. For all organs on T1WI and T2WI (excluding the anal canal), the DSCs of R2 and the automatic segmentation model were both greater than 0.75. On the three sequences, the average DSC values of the observer and the automatic segmentation model were both greater than 0.78, with the highest DSC value (equal to 0.696) observed on T2WI. Therefore, we found T2WI to be the best sequence for automatic segmentation in this study; this result is consistent with the results of another study on MRIbased automatic segmentation of the pelvis.^25^

The sequence used for the automatic segmentation of pelvic tumors in most studies is T2WI because it yields better imaging results for the target area of pelvic tumors. In this study, we compared the automatic segmentation results among the tested sequences and found that T2WI and T1WI yielded the most similar results in the automatic segmentation model (highest DSC and lowest 95% HD). This suggests that T1WI may serve as a substitute sequence for T2WI when using a single sequence for model segmentation. Our results yielded a high similarity in manual delineation and automatic segmentation models between T1WI and T2WI. This may indicate that T1WI can be used as a supplementary information input when constructing automatic segmentation models, as also shown by Chi *et al*. who used a T2-weighted image and segmented the bladder outer wall boundary using a T1-weighted image.^26^

In this study, we analyzed the differences among the three MRI sequences used in radiotherapy and provided a reference for sequence selection in an MR-only workflow. Our research exhibits certain limitations. Initially, our investigation has been confined to assessing the delineation consistency of organs at risk across various imaging modalities. However, the contouring of the target volume is equally pivotal and warrants further exploration in terms of how imaging sequences may affect its delineation. Secondly, this study involved only two observers, which may have influenced the results. In clinical settings, there is inherent variability among practitioners within the same specialty, across different specialties, and even among institutions. To mitigate these biases and enhance the generalizability of our findings, future studies will incorporate a larger cohort of observers and multi-institutional collaborations, thereby aiming to deliver more equitable and evidence-based recommendations.

## Conclusions

In this study, we analyzed the variability in three MR sequences (T1WI, T2WI, and T1dixonc) based on the delineation of pelvic organs performed by human observers and automatic segmentation models. The results indicated that human observers demonstrated better results on T1WI, whereas automatic segmentation models demonstrated better results on T2WI. The difference analysis results among the sequences in manual delineation and automatic segmentation indicated good similarity between T1WI and T2WI. Therefore, T1WI, T2WI, or a combination of T1WI and T2WI can be used for the planning of MR-only radiation therapy. To the best of our knowledge, there are few studies on interobserver variability based on pelvic MR multiple-sequence imaging.

## Supplementary Material

Supplementary Material Details
