# Supplementary material for: Interobserver and sequence variability in the delineation of pelvic organs at risk on magnetic resonance images
Source: Radiol Oncol. 2025 Jan 22;59(1):139–46. doi: 10.2478/raon-2025-0006 (PMC11867573; doi:10.2478/raon-2025-0006)
Supplement: Supplementary file 1 — Supplementary Material Details [file raon-2025-0006_sm.pdf]

Interobserver and sequence variability in the delineation of pelvic organs at risk on magnetic resonance images

Wanjia Zheng, Xin Yang, Zesen Cheng, Jinxing Lian, Enting Li, Shaolin Mo, Yimei Liu, Sijuan Huang

doi: 10.2478/raon-2025-0006

SUPPLEMENTARY TABLE 1. Comparison of the volume of each organ delineated by two observers and auto-segmentation

		Bladder	Rectum	Anal canal	Femoral head _L	Femoral head _R
**Volume(cm3)**						
	T1WI	237.074±132.318	45.463±16.434	7.299±1.620	75.951±12.772	74.035±11.360
R1	T1dixonc	305.863±152.644	48.239±18.382	7.329±1.819	75.778±12.034	75.460±12.089
	T2WI	213.734±125.164	46.598±16.412	9.590±2.668	71.780±12.155	72.197±10.626
	T1WI	237.357±127.505	44.884±14.573	7.407±1.552	80.358±12.151	79.621±10.993
R2	T1dixonc	296.087±146.813	46.393±15.176	7.584±1.717	75.115±12.403	76.751±11.427
	T2WI	225.209±125.934	43.601±12.268	8.273±1.843	80.944±12.236	80.292±10.921
	T1WI	237.919±119.003	39.586±13.126	7.047±1.970	71.209±16.449	70.697±11.696
Auto	T1dixonc	288.749±137.264	29.838±10.975	8.369±2.892	72.994±11.003	76.307±12.378
	T2WI	205.746±121.853	37.329±12.193	9.816±2.492	77.165±12.275	75.199±10.544
**P value**						
	T1WI	0.770	0.888	0.888	0.003	0.001
R1-R2	T1dixonc	0.024	0.443	0.871	0.673	0.170
	T2WI	0.001	0.068	0.048	< 0.001	< 0.001
	T1WI- T1dixonc	< 0.001	0.031	0.888	< 0.001	< 0.001
R2-R2	T1WI- T2WI	< 0.001	0.370	0.006	0.011	0.325
	T2WI- T1dixonc	< 0.001	0.021	0.011	< 0.001	< 0.001
	T1WI	0.538	0.033	0.535	0.083	0.056
R1-Auto	T1dixonc	0.030	< 0.001	0.123	0.088	0.670
	T2WI	0.139	0.001	0.871	0.007	0.035
	T1WI	0.820	0.026	< 0.001	< 0.001	< 0.001
R2-Auto	T1dixonc	0.251	< 0.001	0.179	0.112	0.766
	T2WI	< 0.001	0.004	0.005	< 0.001	< 0.001
	T1WI- T1dixonc	0.001	< 0.001	0.005	0.567	< 0.001
Auto-Auto	T1WI- T2WI	< 0.001	0.041	< 0.001	< 0.001	< 0.001
	T2WI- T1dixonc	< 0.001	< 0.001	0.002	0.001	0.405

L = left; R = right; R1 and R2 = two observers, pelvic oncologists; T1dixonc = contrast enhanced Dixon T1-weighted; T1WI = T1-weighted; T2WI = T2-weighted

SUPPLEMENTARY TABLE 2. Intraclass correlation coefficients (ICCs) for the volume of organs at risk (OARs) between different sequences for observer R2

	T1WI-T1dixonc	T1WI–T2WI	T2WI–T1dixonc
**OARs**	**ICC (95% CI)**	**ICC (95% CI)**	**ICC (95% CI)**
Bladder	0.833 (0.291–0.943)	0.992 (0.907–0.998)	0.788 (0.127–0.930)
	p < 0.001	p <0.001	p <0.001
Rectum	0.965 (0.921–0.984)	0.948 (0.891–0.975)	0.892 (0.757–0.951)
	p < 0.001	p <0.001	p <0.001
Anal canal	0.660 (0.393–0.824)	0.614 (0.236–0.814)	0.418 (0.086–0.672)
	p < 0.001	p <0.001	p =0.007
Femoral head _L	0.894 (0.001–0.975)	0.993 (0.983–0.997)	0.873 (-0.019–0.969)
	p <0.001	p <0.001	p <0.001
Femoral head _R	0.935 (0.612–0.979)	0.988 (0.972–0.995)	0.909 (0.446–0.972)
	p < 0.001	p < 0.001	p < 0.001

CI: confidence interval; L = left; R = right; T1dixonc = contrast enhanced Dixon T1-weighted; T1WI = T1-weighted; T2WI = T2-weighted

SUPPLEMENTARY TABLE 3. Intraclass correlation coefficients (ICC) between automatic segmentation and manual contour volumes delineated by different observers (R1, R2) in three sequences

		T1WI		T1dixonc		T2WI	
	**OARs**	**ICC (95%CI)**	**P**	**ICC (95%CI)**	**P**	**ICC (95%CI)**	**P**
	Bladder	0.933 (0.863~0.968)	<0.001	0.956 (0.900~0.980)	<0.001	0.983 (0.962~0.992)	<0.001
	Rectum	0.517 (0.198~0.738)	0.001	0.272 (-0.097~0.593)	0.005	0.446 (0.069~0.704)	0.001
R1-Auto	Anal canal	0.028 (-0.085~0.591)	0.063	0.002 (-0.037~0.077)	0.468	0.026 (-0.353~0.390)	0.447
	Femoral head _L	0.517 (0.203~0.737)	0.001	0.715 (0.478~0.855)	<0.001	0.616 (0.285~0.807)	<0.001
	Femoral head _R	0.673 (0.444~0.844)	<0.001	0.631 (0.348~0.809)	<0.001	0.694 (0.440~0.844)	<0.001
	Bladder	0.937 (0.871~0.970)	<0.001	0.971 (0.941~0.986)	<0.001	0.975 (0.823~0.992)	<0.001
	Rectum	0.581 (0.274~0.780)	<0.001	0.245 (-0.097~0.561)	0.009	0.548 (0.191~0.768)	<0.001
R2-Auto	Anal canal	0.510 (0.188~0.734)	0.002	0.166 (-0.192~0.492)	0.184	0.345 (-0.004~0.624)	0.010
	Femoral head _L	0.534 (0.108~0.773)	<0.001	0.816 (0.645~0.909)	<0.001	0.909 (0.492~0.971)	<0.001
	Femoral head _R	0.645 (-0.069~0.881)	<0.001	0.783 (0.587~0.892)	<0.001	0.837 (0.106~0.952)	<0.001

OARs = organs at risk; T1dixonc = contrast enhanced Dixon T1-weighted; T1WI = T1-weighted; T2WI = T2-weighted

SUPPLEMENTARY TABLE 4. Comparison of two observers, R1 and R2, with automatic segmentation (Mean ± SD)

		R1-automatic segmentation			R2-automatic segmentation		
	OARs	T1WI	T1dixonc	T2WI	T1WI	T1dixonc	T2WI
	Bladder	0.877±0.192	0.882±0.181	0.891±0.182	0.878±0.192	0.884±0.180	0.891±0.183
	Rectum	0.739±0.185	0.664±0.177	0.725±0.172	0.750±0.189	0.673±0.189	0.763±0.177
**DSC**	Anal canal	0.662±0.162	0.647±0.172	0.669±0.157	0.649±0.163	0.647±0.186	0.696±0.153
	Femoral head _L	0.856±0.182	0.876±0.164	0.886±0.166	0.881±0.193	0.890±0.167	0.922±0.172
	Femoral head _R	0.878±0.168	0.866±0.163	0.900±0.163	0.881±0.171	0.893±0.168	0.915±0.171
	Averaged DSC	0.802±0.130	0.787±0.140	0.815±0.122	0.809±0.141	0.796±0.149	0.838±0.115
	Bladder	10.695±11.041	10.631±10.463	6.900±5.718	10.577±10.939	10.906±0.654	6.641±5.916
	Rectum	12.589±10.885	10.877±6.362	11.238±6.499	10.655±10.383	9.668±4.817	7.945±4.674
**95% HD**	Anal canal	5.596±2.713	6.308±3.074	6.548±2.426	5.911±2.664	6.212±2.771	5.166±1.981
**(mm)**	Femoral head _L	5.047±2.432	4.611±1.866	5.386±2.290	4.304±1.618	4.314±1.618	3.198±1.193
	Femoral head _R	4.890±1.901	6.292±5.230	4.208±1.855	4.336±2.025	4.751±4.263	3.172±1.036
	Averaged 95% HD	7.763±7.781	7.744±6.593	6.856±4.810	7.157±7.509	7.170±6.260	5.224±3.984

DSC = dice similarity coefficient; HD = Hausdorff distance; T1dixonc = contrast enhanced Dixon T1-weighted; T1WI = T1-weighted; T2WI = T2-weighted

SUPPLEMENTARY TABLE 4. Intraclass correlation coefficients (ICC) for the volume between the different sequences for automatic segmentation

	T1WI-T1dixonc		T1WI -T2WI		T2WI-T1dixonc	
OARs	ICC (95%CI)	P	ICC (95%CI)	P	ICC (95%CI)	P
Bladder	0.807 (0.424~0.924)	< 0.001	0.909 (0.660~0.966)	<0.001	0.754 (-0.040~0.927)	< 0.001
Rectum	0.565 (-0.039~0.826)	< 0.001	0.897 (0.778~0.952)	<0.001	0.632 (0.083~0.849)	< 0.001
Anal canal	0.539 (0.173~0.764)	< 0.001	0.377 (-0.106~0.718)	<0.001	0.588 (0.200~0.799)	< 0.001
Femoral head _L	0.637 (0.361~0.811)	< 0.001	0.696 (0.374~0.856)	<0.001	0.834 (0.495~0.935)	< 0.001
Femoral head _R	0.779 (0.285~0.917)	< 0.001	0.852 (0.314~0.951)	<0.001	0.871 (0.747~0.937)	< 0.001
